# A new predictive factor VGF based on IHC experiments, gene pathways and molecular functional groups for tumor immune microenvironment and prognosis of adrenocortical carcinoma

**DOI:** 10.3389/fimmu.2025.1542780

**Published:** 2025-04-17

**Authors:** Junpeng Chi, Keyuan Lou, Jiankun Zhang, Jitao Wu, Yuanshan Cui

**Affiliations:** ^1^ Department of Urology, The Affiliated Yantai Yuhuangding Hospital of Qingdao University, Yantai, China; ^2^ School of Clinical Medicine, Shandong Second Medical University, Weifang, China; ^3^ Department of Urology, Weifang People’s Hospital, Weifang, China

**Keywords:** VGF nerve growth factor inducible, adrenocortical carcinoma, gene expression, immune microenvironment, survival prognosis analysis

## Abstract

**Background:**

Adrenocortical carcinoma (ACC) is a rare and aggressive malignancy with a poor prognosis, and its clinical management remains a significant challenge due to the high recurrence rates and limited treatment options. Despite advances in understanding the molecular mechanisms underlying ACC, no reliable biomarkers have been validated for routine clinical use.

**Methods:**

We analyzed RNA sequencing data from The Cancer Genome Atlas (TCGA) database (n=79) and Genotype Tissue Expression (GTEx) database (n=128) to investigate the expression of VGF in ACC and normal adrenal tissues. Gene expression levels of VGF were quantified and correlated with clinicopathological features and survival outcomes. Statistical methods included Cox proportional hazards models and Kaplan-Meier analysis, while Gene Set Enrichment Analysis (GSEA) was utilized to identify relevant biological pathways associated with VGF expression. Clinical data from 7 ACC patients from YANTAI YUHUANGDING Hospital were also analyzed. The expression of VGF in ACC and normal adrenal gland tissue was further validated through IHC experiments.

**Results:**

Our results demonstrate that VGF expression is elevated in ACC tissues compared to normal adrenal tissues and is significantly associated with advanced disease stages, lymph node involvement, metastasis and poor overall survival. VGF levels also correlate with immune cell infiltration, including Th2 cells, T helper cells, and Neutrophils. Importantly, our study establishes VGF as a potential prognostic biomarker for ACC and highlights its role in tumor progression and immune modulation. Additionally, GSEA analysis suggests that VGF is involved in cytokine receptor interaction and the P13K-Akt signaling pathway, possibly relating to tumor immunity.

**Conclusions:**

VGF could serve as a valuable marker for patient stratification, monitoring disease progression, and predicting responses to immunotherapies. Future studies should focus on investigating circulating VGF levels as a non-invasive biomarker for ACC to improve clinical management and treatment outcomes.

## Background

Adrenocortical carcinoma (ACC) is a rare and aggressive cancer originating from the adrenal cortex. ACC has a poor prognosis and requires better diagnostic and treatment approaches due to its late detection, fast progression, and limited response to standard therapies (1, 2). ACC is characterized by high clinical and genetic heterogeneity, contributing to the variable prognosis and therapeutic responses observed among patients (3). Typically, the disease manifests with symptoms resulting from excessive hormone production or as incidental findings on imaging. The current five-year survival rate for ACC patients is approximately 40%, but this drops sharply with advanced disease stage or metastasis at diagnosis. Surgical resection remains the primary treatment modality, supplemented by adjuvant therapies in cases with a high risk of recurrence (4, 5). However, the effectiveness of available chemotherapeutic options is limited, and recurrence is common, leading to a dismal prognosis for many patients (6, 7).

Recent research has highlighted the potential role of various biomarkers in predicting disease progression and therapeutic outcomes in ACC (8, 9). Although several biomarkers have been investigated for their potential roles in ACC, there is currently no validated biomarker for routine clinical use. Several candidate biomarkers have been explored, including cortisol levels, aldosterone levels and steroidogenic enzymes (10, 11). These biomarkers are primarily useful in identifying functioning ACC, but their prognostic value for non-functioning tumors is limited. VGF nerve growth factor inducible (VGF, not an acronym) is a secreted polypeptide that plays a role in metabolic regulation. It is induced by neurotrophic factors and is involved in neurite growth and neuroprotection (12, 13). VGF or its derived peptides could potentially serve as biomarkers or targets for therapy in various conditions including obesity, dementia, depression, and pain (14). VGF is a gene product that serves as a precursor for various neuro-endocrine peptides and disease biomarkers (15). Its role in malignant tumors has not been extensively studied. However, some research suggests that the peptide products of the VGF are generated in human neuroendocrine cells during early development and their production increases in cases of hyperplasia and neoplasia (16). This has prompted our interest in investigating the connection between VGF and neuroendocrine tumors.

The exploration of VGF in ACC has been limited. Thus, the objective of this study is to evaluate the prognostic value of VGF expression in ACC based on data obtained from TCGA. We investigated the expression levels of VGF and correlated it with survival in ACC patients. Through immune infiltration analysis and gene enrichment analysis network, we further investigated the important role of VGF in the immune microenvironment and cell cycle. This research seeks to determine whether VGF expression levels can serve as a prognostic biomarker in ACC. In addition, through the analysis of related biological pathways, this study may identify possible mechanisms through which VGF influences the development of ACC. It is expected to provide some guidance for the determination of new targets for precise treatment of ACC.

## Materials and methods

### Data sources

We used 128 normal adrenal tissue samples from the GTEx database (https://xenabrowser.net/datapages/). A total of 77 ACC cases with gene expression data were collected from TCGA (https://portal.gdc.cancer.gov/). Samples with RNA-seq data that lacked corresponding clinical data were excluded from the analysis. After filtering, the RNA-Seq gene expression TPM (transcripts per million reads) of 79 cases with ACC and clinical data were retained and further analyzed. The clinical characteristics of ACC patients are shown in **Supplementary Figure 1**. The HTSeq-FPKM data were transformed to TPM (transcription per million reads) for the following analyses.

Clinical data mainly included age, sex, stage, grade, TNM stage, survival status and survival time. VGF expression levels in ACC tissues from the TCGA database were categorized as either VGF-high or VGF-low based on whether the values were above or below the median value of VGF. In addition, the gene expression data was normalized by log2 (TPM+1) for later data analysis.

ACC tissue and normal tissue(> 2 cm adjacent to the cancer) were taken from 7 surgically removed ACC specimens at YANTAI YUHUANGDING Hospital. This study was conducted with the approval of the institutional ethics board of the YANTAI YUHUANGDING Hospital.

### Analysis of differentially expressed genes for ACC between high and low VGF expression groups

DEGs were identified by comparing the expression profiles (HTSeq-Counts) of the high VGF expression group and the low VGF expression group using the Wilcoxon rank-sum test in R language-related software, “DESeq2 and EdgeR” (17). Differences with a |log_2_fold change (FC)|>2 and adjusted P-value (p.adj5) < 0.05 were considered as threshold values for identifying DEGs.

### Gene set enrichment analysis

Assessing the distribution trends of genes in phenotype-associated gene expression data in GESA (https://www.gsea-msigdb.org/gsea/msigdb/collections.jsp) to determine their contribution and correlation with the phenotype (18). Use VGF as a grouping method to explore gene enrichment related to it, including pathways and molecular functions. Gene Ontology (GO) and Kyoto Encyclopedia of Genes and Genomes (KEGG) enrichment analyses were performed using the “clusterProfiler and ggplot2” package in R software (19, 20). The significant criteria are typical: p.adj < 0.05 and FDR(qvalue) < 0.25.

### DNA methylation of the VGF gene

DNA methylation has critical implications in predicting prognosis and serving as a potential biomarker in the development and advancement of tumors. To investigate the relationship between the expression and prognostic patterns of a specific CpG methylation site in the VGF gene in ACC, we utilized MethSurv (https://biit.cs.ut.ee/methsurv/), an online tool that integrates various analyses related to DNA methylation (21, 22). The DNA methylation results of VGF were generated via the MethSurv platform.

### Immune infiltration analysis by ssGSEA

The immune infiltration analysis of ACC was done by ssGSEA (single-sample gene Set EnrichmentAnalysis) method using “GSVA” package in R for 24 types of immune cells in tumor samples. The correlation between VGF expression and markers of 24 immune cell types described previously was validated using “ggplot2 and ggalluvial” package in R. The correlation between VGF and these immune cells was analyzed by Spearman correlation, and the infiltration of immune cells between the high and low-expression groups of VGF was analyzed by the Wilcoxon rank sum test.

### Immunohistochemistry staining and analysis

Adrenal tissue paraffin-embedded samples were processed into 5-μm-thick slices after 10% formalin fixation. The paraffin sections were deparaffinized and rehydrated. Tissue blocks were then treated with 3% hydrogen peroxide and blocked using 3% bovine serum albumin (BSA). After blocking, the sections were incubated with primary antibody (D162477, VGF Antibody, in dilution 1:50; Sangon Biotech shanghai, China) overnight at 4°C. Sections were incubated with secondary antibody (D110058, Goat anti rabbit lgG at 1/200 dilution; Sangon Biotech shanghai, China) and stained with DAB kit (ZSGB-bio, China) and hematoxylin the following day.

The percentages for grading staining are as follows: 0 for no staining, 1 for 1-10% staining, 2 for 11-49% staining, and 3 for 50-100% staining. Staining intensity is scored as 0 for negative, 1 for weak, 2 for moderate, and 3 for strong. The expression level of VGF is analyzed as VGF-low (IHC score<6) and VGF-high (IHC score ≥ 6). Three independent observers inspected the specimens in a blinded manner.

### Statistical analysis

Statistical analyses in R (v.4.2.1) were used to assess the relationship between clinical pathologic features and VGF. The Wilcoxon signed-rank test and logistic regression were employed for analysis. Cox regression and the Kaplan-Meier method were used to examine clinicopathologic characteristics associated with overall survival in TCGA patients. Multivariate Cox analysis compared the impact of VGF expression on survival along with other clinical characteristics (pathologic stage, clinical stage, residual tumor, lymph node status, distant metastasis status and age). The median value determined the cut-off value of VGF expression. Cox regression and nomograms were constructed using the “survival and rms” packages. Statistical significance was considered at P<0.05.

### Ethical statement

The Ethical Committee of YANTAI YUHUANGDING Hospital approved this study (No. 2024-384). All samples and clinical information used in this study were obtained with informed consent from patients.

## Results

### Pan-cancer analysis of VGF expression

**Figure 1:**
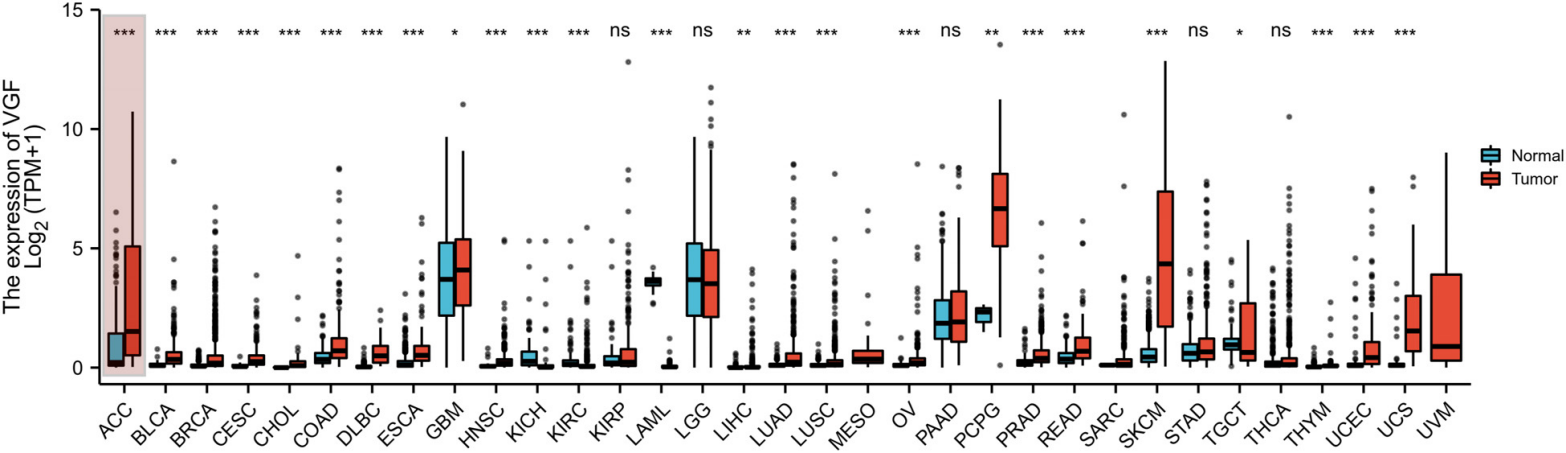
Expression pattern of VGF in a pan-cancer perspective. The mRNA expression of VGF is commonly increased in most of the 33 cancer types, while it is decreased in a few of them as compared to normal tissues. (ns: P ≥0.05; *: P <0.05; **: P <0.01; ***: P <0.001) (ACC, Adrenocortical Carcinoma; BLCA, Bladder Urothelial Carcinoma; BRCA, Breast Invasive Carcinoma; CESC, Cervical Squamous Cell Carcinoma and Endocervical Adenocarcinoma; CHOL, Cholangiocarcinoma; COAD, Colon Adenocarcinoma; DLBC, Diffuse Large B-Cell Lymphoma; ESCA, Esophageal Carcinoma; GBM, Glioblastoma Multiforme; HNSC, Head and Neck Squamous Cell Carcinoma; KICH, Kidney Chromophobe; KIRC, Kidney Renal Clear Cell Carcinoma; KIRP, Kidney Renal Papillary Cell Carcinoma; LAML, Acute Myeloid Leukemia; LGG, Lower Grade Glioma; LIHC, Liver Hepatocellular Carcinoma; LUAD, Lung Adenocarcinoma; LUSC, Lung Squamous Cell Carcinoma; MESO, Mesothelioma; OV, Ovarian Serous Cystadenocarcinoma; PAAD, Pancreatic Adenocarcinoma; PCPG, Pheochromocytoma and Paraganglioma; PRAD, Prostate Adenocarcinoma; READ, Rectum Adenocarcinoma; SARC, Sarcoma; SKCM, Skin Cutaneous Melanoma; STAD, Stomach Adenocarcinoma; TGCT, Testicular Germ Cell Tumors; THCA Thyroid Carcinoma; THYM, Thymoma; UCEC, Uterine Corpus Endometrial Carcinoma; UCS, Uterine Carcinosarcoma; UVM, Uveal Melanoma.

The expression levels of VGF across various cancer types, both normal and tumor tissues, are presented in the plot (**Figure 1**). The statistical analysis showed significant overexpression of VGF in tumor tissues for most cancer types. VGF is markedly upregulated in tumors such as ACC (Adrenocortical Carcinoma), BLCA (Bladder Urothelial Carcinoma), BRCA (Breast Cancer), and others. These findings suggest a potential correlation between elevated VGF levels and tumors.

### VGF Expression was significantly elevated in ACC tissues

**Figure 2:**
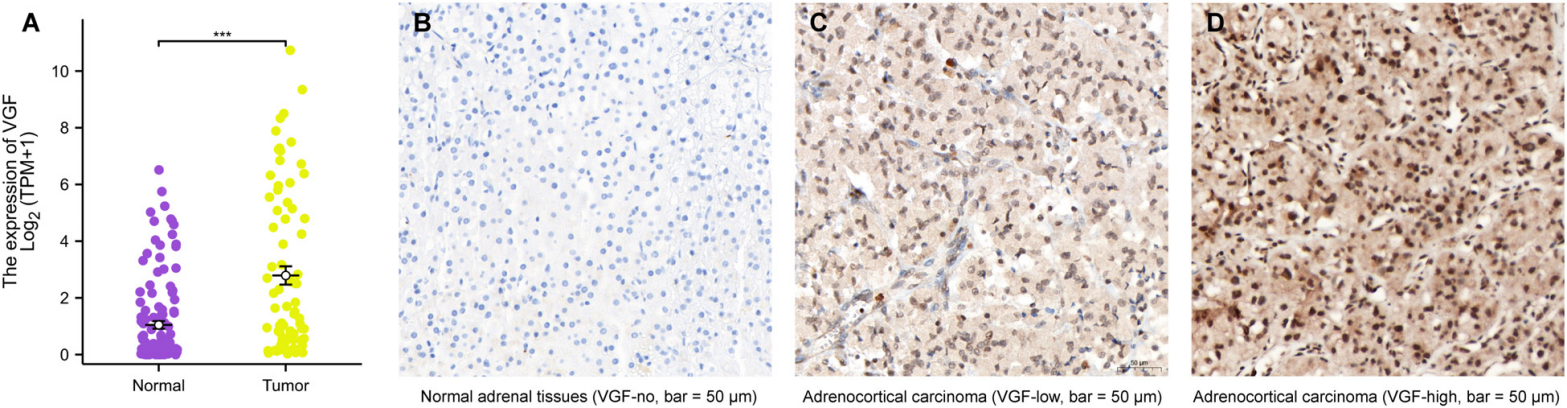
The mRNA and protein expression of VGF in ACC. **(A)** mRNA expression levels of VGF in 77 ACC samples and 128 normal samples. Immunohistochemical staining results of VGF in normal adrenal tissues (**B**: VGF-no) and ACC samples (**C**: VGF-low, **D**: VGF-high) (bar = 50 μm). (^∗∗∗^P <0.001).

A box plot comparing the mRNA expression of VGF between normal adrenal and tumor tissues shows significant upregulation of VGF in tumors. The statistical analysis confirms that VGF expression is significantly higher in tumor tissues (p < 0.001), suggesting its potential as a biomarker for cancer. Tumor samples generally exhibit higher VGF expression levels, as seen from the spread of the data points (**Figure 2A**). In addition, VGF mRNA expression was significantly elevated in ACC tissues compared to normal tissues, as determined using the Gepia website (http://gepia2.cancer-pku.cn/#analysis, *: p < 0.05, **Supplementary Figure 1**).

Clinical information was collected from ACC patients who had surgery at YANTAI YUHUANGDING Hospital from January 2011 to January 2022. The normal adrenal cortex tissues are arranged neatly into different zones, while the ACC tissues show disordered arrangement and heterogeneity, with large and deeply stained nuclei. Additionally, immunohistochemical staining revealed higher protein expression of VGF in ACC samples compared to normal adrenal tissues, as depicted in **Figures 2B–D**.

### DNA methylation analysis of the VGF gene in ACC

**Figure 3:**
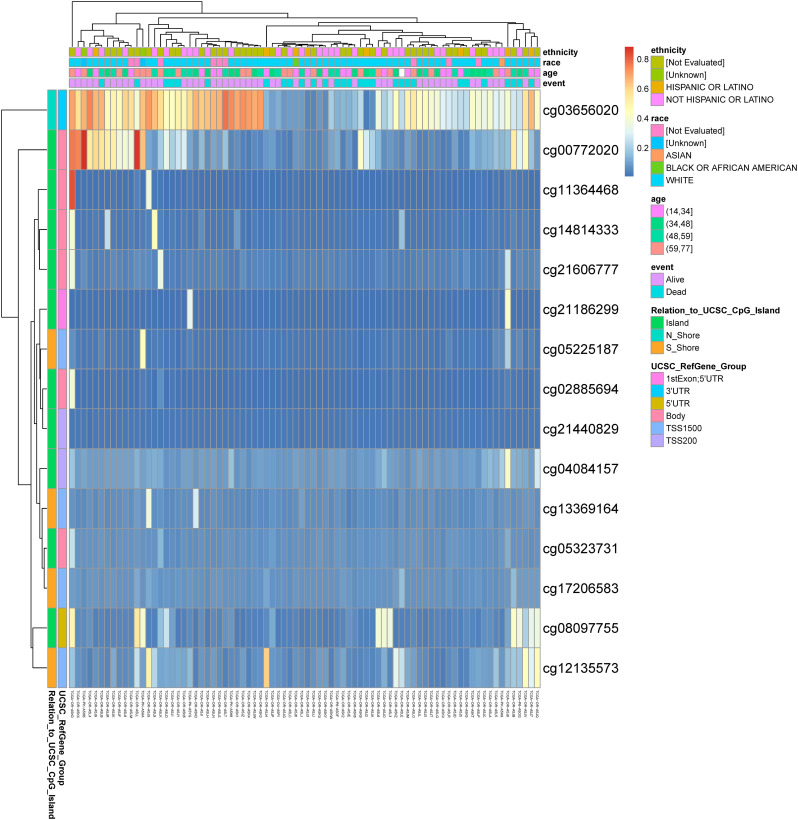
The DNA methylation of VGF in ACC of TCGA. Heatmap of DNA methylation expression levels of the VGF gene in ACC by MethSurv platform.

DNA methylation is a crucial epigenetic modification that can influence gene expression and play a significant role in cancer development and progression. In this study, we analyzed the DNA methylation status of the VGF gene in ACC. We showed the heatmap of DNA methylation clustering the expression levels of the VGF gene in ACC. **Figure 3** illustrates a heatmap of DNA methylation levels at various CpG sites across the VGF gene locus. The samples are grouped based on their clinical characteristics, including ethnicity, race, age, and event (alive or dead). This suggests that aberrant methylation of the VGF gene may be associated with ACC pathogenesis.

### VGF mRNA levels are associated with clinicopathological characteristics in ACC patients

**Figure 4:**
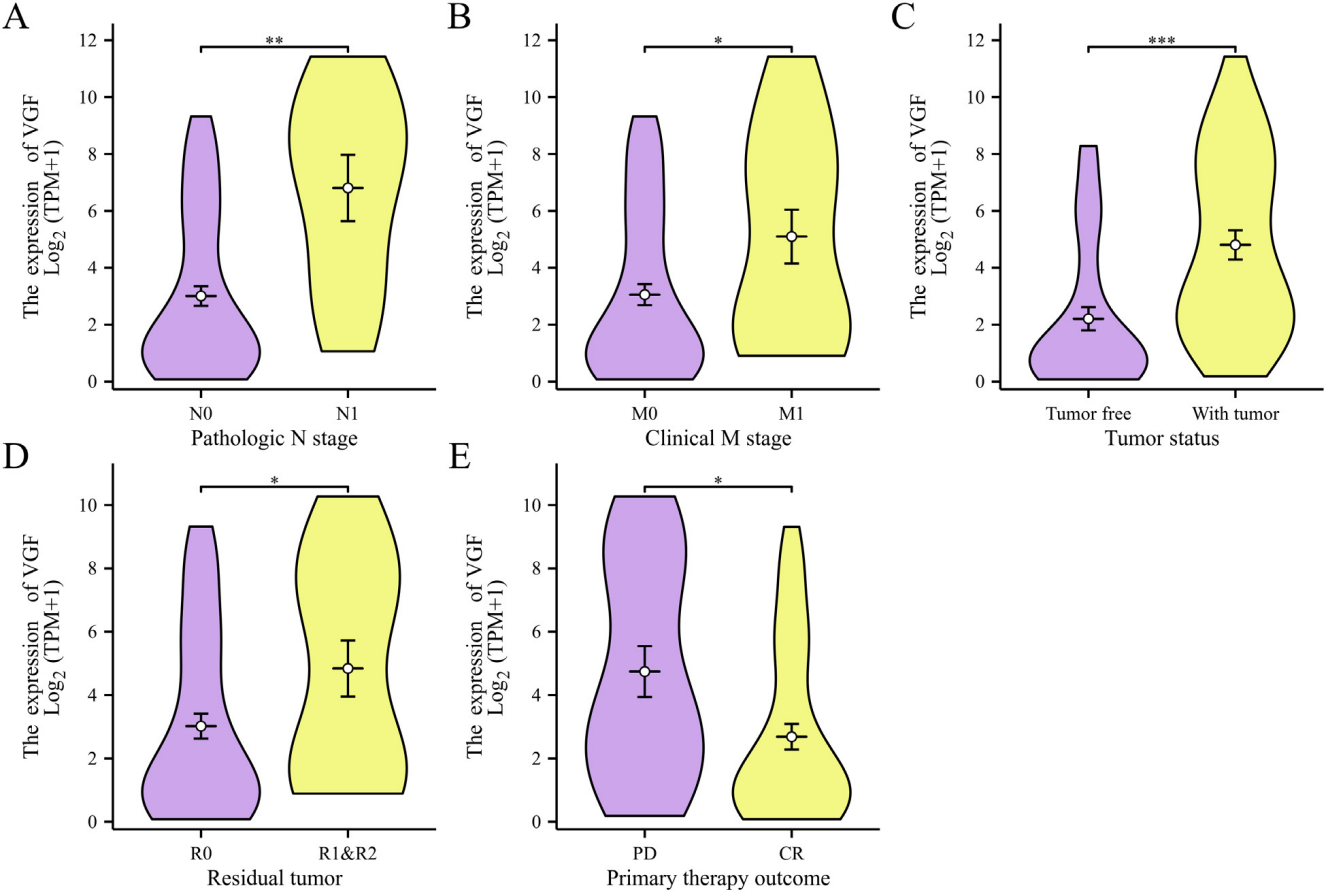
Association with VGF expression and clinicopathological characteristics, including N stages **(A)**, M stages **(B)**, tumor status **(C)**, residual tumor **(D)** and primary therapy outcome **(E)**. (PD, Progressive disease; CR, Complete response; ^∗^P <0.05; ^∗∗^P <0.01; ^∗∗∗^P <0.001).

Dunn’s tests and Kruskal-Wallis tests were performed to evaluate the relationship between VGF mRNA expression and clinical pathological characteristics of ACC patients. As shown in **Figure 4**, higher levels of VGF expression demonstrated a significantly positive correlation with the N stages (N1 vs. N0, p < 0.01) and M stages (M1 vs. M0, p < 0.05). Increased VGF expression is also correlated with tumor status (with tumor vs. tumor free, p < 0.001), residual tumor (R1&R2 VS. R0, p < 0.05) and primary therapy outcome (PD VS. CR, p < 0.05). These outcomes suggested that VGF may have a role as a biomarker of poor prognosis in ACC and is a potential therapeutic target for ACC.

### Univariate and multivariate cox regression analysis

**Figure 5:**
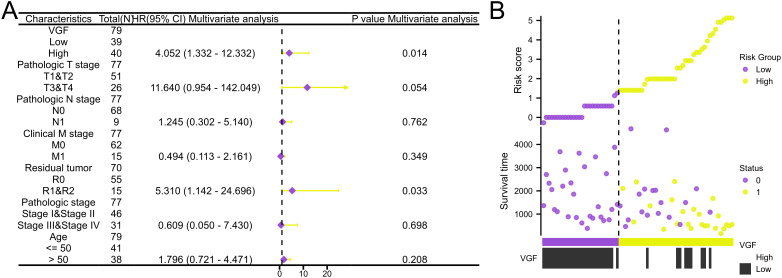
**(A)** Forest plot: Multivariate Cox analysis of VGF expression and other clinicopathological variables. **(B)** Risk Factor Plot: Risk Score and Survival Time of VGF Expression Distribution in Low and High Expression Groups (0, Alive; 1, dead).

We analyzed the correlation between overall survival and multivariable characteristics in ACC. Univariate analysis showed that VGF expression (HR = 4.071, p = 0.002), T stage (HR = 10.286, p < 0.001), M stage (HR = 6.150, p < 0.001), Residual tumor (HR = 12.617, p < 0.001) and pathologic stage (HR = 6.476, p < 0.001) are significantly correlated with OS (**Table 1**). In multivariate analysis, only VGF expression (HR = 4.052, p = 0.014) and Residual tumor (HR = 5.310, p = 0.033) were identified as independent prognostic factors for ACC. Using multivariate analysis, the data revealed that VGF expression (HR = 4.052, p = 0.014) is an independent factor for prognosis (**Figure 5A**, **Table 1**). The distribution of VGF expression, survival status and risk score of ACC patients were analyzed, as shown in **Figure 5B**. The analysis also revealed that the low-risk group had lower levels of VGF expression and better survival compared to the high-risk group.

**Table 1:** Univariate and multivariate Cox proportional hazards analyses of VGF expression and OS for patients with ACC.

Characteristics	Total(N)	Univariate analysis		Multivariate analysis	
		Hazard ratio (95% CI)	P value	Hazard ratio (95% CI)	P value
**VGF**	79				
Low	39				
High	40	4.071 (1.645 - 10.074)	**0.002**	4.052 (1.332 - 12.332)	**0.014**
**Pathologic T stage**	77				
T1&T2	51				
T3&T4	26	10.286 (3.976 - 26.608)	**< 0.001**	11.640 (0.954 - 142.049)	0.054
**Pathologic N stage**	77				
N0	68				
N1	9	2.038 (0.769 - 5.400)	0.152	1.245 (0.302 - 5.140)	0.762
**Clinical M stage**	77				
M0	62				
M1	15	6.150 (2.710 - 13.959)	**< 0.001**	0.494 (0.113 - 2.161)	0.349
**Residual tumor**	70				
R0	55				
R1&R2	15	12.617 (5.064 - 31.434)	**< 0.001**	5.310 (1.142 - 24.696)	**0.033**
**Pathologic stage**	77				
Stage I&Stage II	46				
Stage III&Stage IV	31	6.476 (2.706 - 15.498)	**< 0.001**	0.609 (0.050 - 7.430)	0.698
**Age**	79				
<= 50	41				
> 50	38	1.799 (0.846 - 3.824)	0.127	1.796 (0.721 - 4.471)	0.208

The values shown in bold in the table are statistically significant.

### VGF expression is associated with survival time and prognostic value in ACC patients

**Figure 6:**
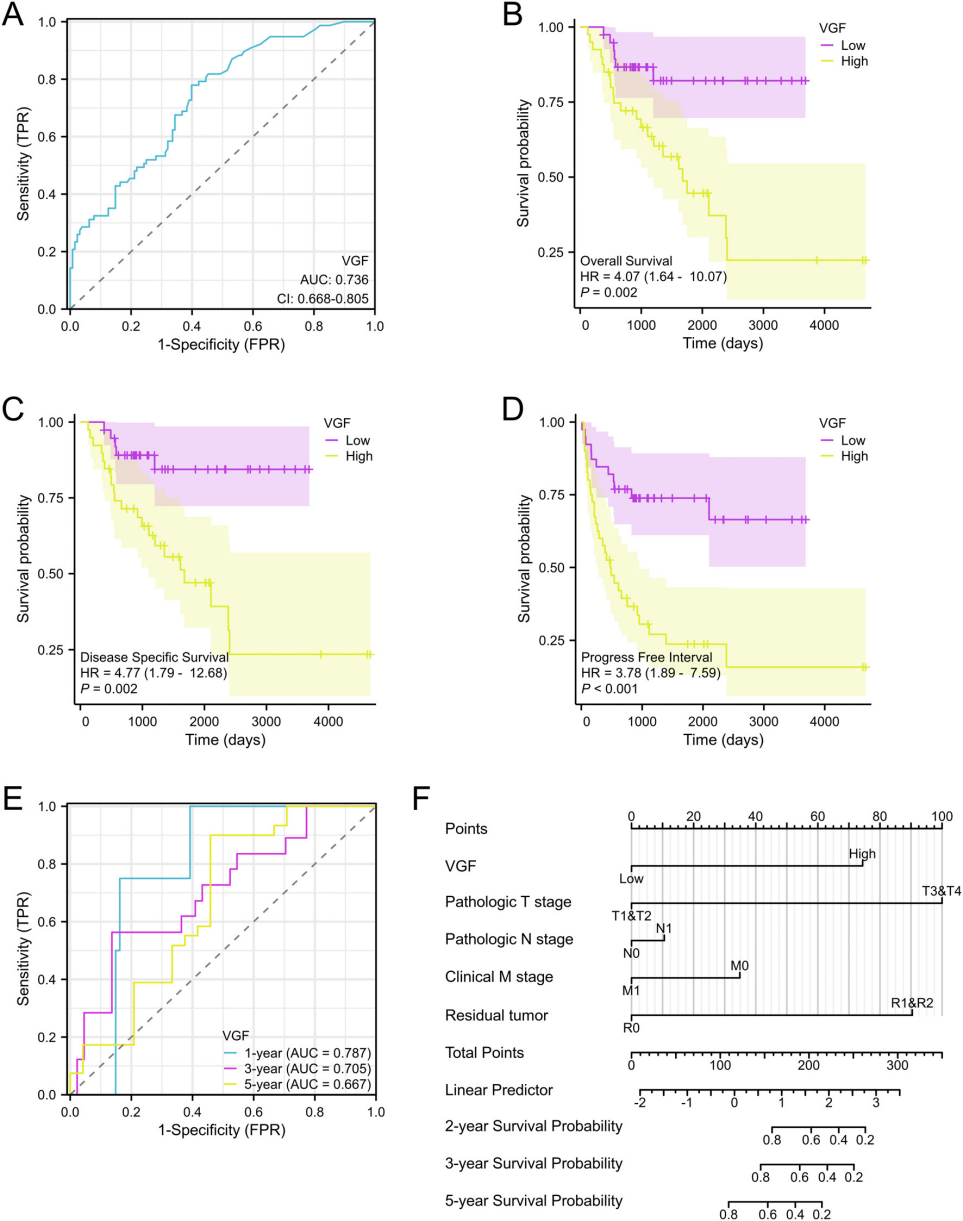
ROC and Kaplan-Meier curves for VGF. **(A)** ROC curve showed that VGF had an AUC value of 0.736. **(B-D)** ACC patients with low VGF mRNA expression had longer OS, DSS, and PFI compared to those with high levels of VGF, as indicated by Kaplan-Meier survival curves. **(E)** Time-dependent ROC Curves showed that VGF had an AUC value of 0.787 (1-year), 0.705 (3-year) and 0.667 (5-year). **(F)** Nomogram for predicting the probability of 1-, 3-, and 5-year OS for ACC patients. (OS, overall survival; DSS, disease specific disease; PFI: progress free interval).

The area under the survival curve (AUC) reaches 0.736, which means VGF has a good predictive ability for the diagnosis and prognosis of ACC patients (**Figure 6A**). The results of the Kaplan-Meier survival analysis indicated that ACC patients with a high level of VGF expression were associated with a poorer prognosis in comparison to patients with a low level of VGF expression. Most ACC patients with low VGF expression are in a survival state. **Figures 6B–D** show that the OS (HR = 4.07, 95% CI 1.64 - 10.07, p = 0:002), DSS (HR = 4.77, 95% CI 1.79 - 12.68, p = 0:002) and PFI (HR = 3.78, 95% CI 1.89 – 7.59, p < 0.001) of ACC patients that had a high level of VGF were significantly shorter compared to those of a low level of VGF. VGF had AUC values of 0.787 (1-year), 0.705 (3-year), and 0.667 (5-year) based on Time-Dependent ROC Curves (**Figure 6E**). These findings indicate that the VGF expression level can influence the prognosis of ACC patients.

In addition, we developed a nomogram to predict the 2- and 5-year survival based on the prognostic value of VGF in ACC (**Figure 6F**). Based on the clinical and pathological features of the ACC patients, we include TNM staging and residual tumor among these parameters in the predictive model. Believe in the benefits of a useful quantitative model in aiding clinicians to accurately determine the prognosis of ACC patients.

### Identifying DEGs and functional enrichment analysis in high and low VGF expression groups

**Figure 7:**
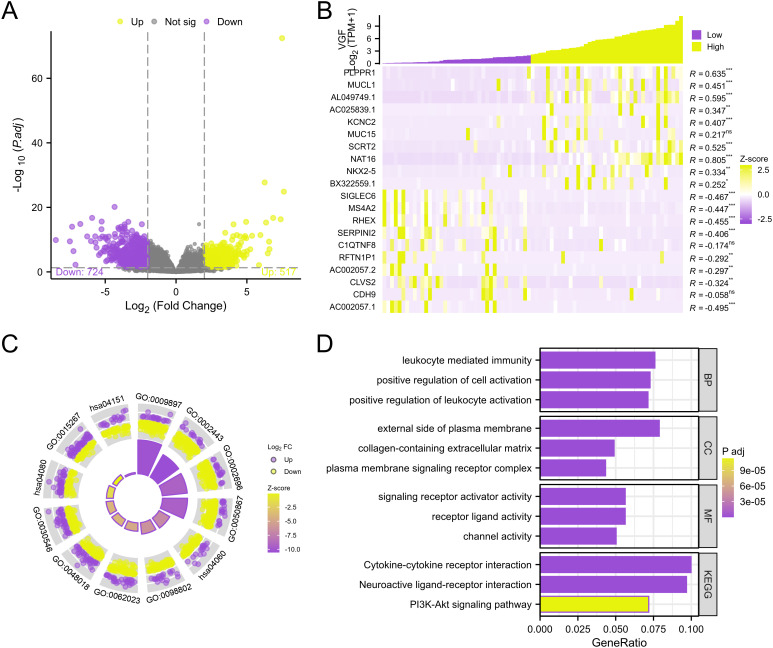
Genes expression correlated with VGF in ACC. **(A)** Volcano plot of differentially expressed genes. **(B)** Heat map of the 20 differentially expressed RNAs, including 10 up-regulated genes and 10 down-regulated genes. **(C, D)** Pathway enrichment results for differentially expressed genes between high and low expression of VGF in ACC patients from KEGG and GO analysis (MF: Molecular Function; CC: Cellular Component; BP: Biological Process; KEGG: Kyoto Encyclopedia of Genes and Genomes; GO: Gene Ontology). (ns: P ≥0.05; *: P <0.05; **: P <0.01; ***: P <0.001).

A total of 1241 genes (including 517 upregulated and 724 downregulated genes) were identified as DEGs between the high VGF and low VGF groups, as shown in the volcano plot (**Figure 7A**). According to the volcano plot, we selected 20 genes (including 10 up-regulated genes and 10 down-regulated genes) based on the values of logFC. We performed a heatmap analysis of DEGs. **Figure 7B** shows the heatmap of the 20 DEGs in the high-level and low-level VGF expression groups. To further understand the biological functions associated with VGF involvement in ACC development, we performed a bar and circle graph analysis of GO-KEGG co-enrichment with logFC (**Figures 7C, D**). Based on the GeneRatio involved in biological pathways, we compared four ontologies: Molecular Function, Cellular Component, Biological Process, and Kyoto Encyclopedia of Genes and Genomes. For each ontology, we selected the top 5 processes based on GeneRatio and generated a table showing the correlation between KEGG and GO terms with VGF expression (**Supplementary Table 2**).

**Figure 8:**
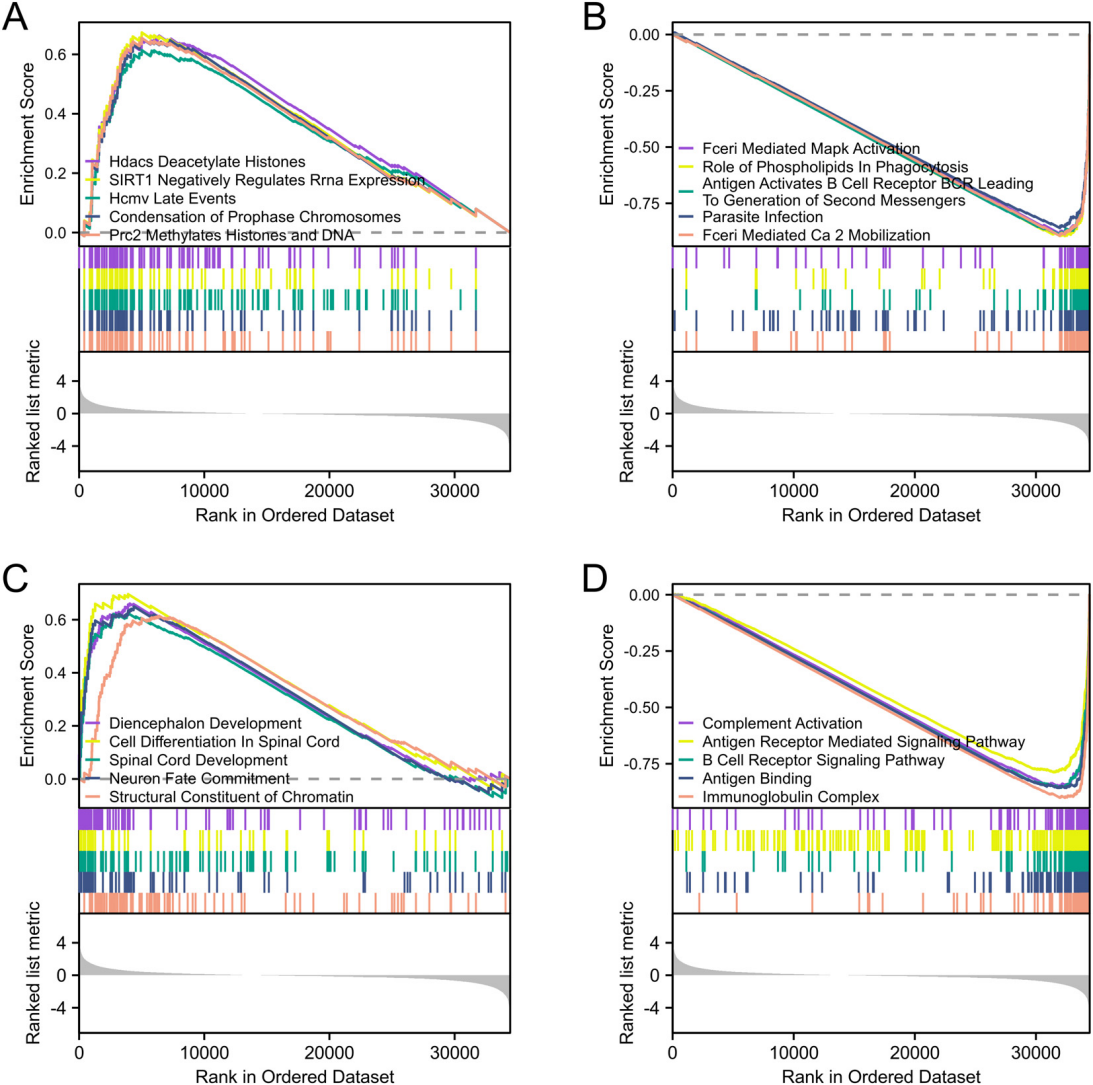
GSEA revealed the top five positively correlated **(A)** and top five negatively correlated groups **(B)** in the KEGG pathway. GSEA revealed the top five positively correlated **(C)** and top five negatively correlated groups **(D)** in the GO term. (GSEA, Gene Set Enrichment Analysis).

GSEA was performed to identify the potential biological function of VGF. GSEA revealed significant differences in the enrichment of GO terms and KEGG pathways in samples between high and low levels of VGF. We selected the most highly enriched signaling pathways based on their normalized enrichment score (NES). In the KEGG pathway enrichment analysis, we selected the five pathways that had the strongest positive correlation with VGF expression: Hdacs Deacetylate Histones, Sirt1 Negatively Regulates Rrna Expression, Hcmv Late Events, Condensation Of Prophase Chromosomes and Prc2 Methylates Histones And Dna (**Figure 8A**, **Table 2**). The five pathways with the strongest negative correlation were Fceri Mediated Mapk Activation, Role Of Phospholipids In Phagocytosis, Antigen Activates B Cell Receptor Bcr Leading To Generation Of Second Messengers, Parasite Infection, Fceri Mediated Ca 2 Mobilization (**Figure 8B**, **Table 2**). GO annotation uncovered five categories that were positively correlated with high levels of VGF: Diencephalon Development, Cell Differentiation in Spinal Cord. Spinal Cord Development, Neuron Fate Commitment and Structural Constituent of Chromatin (**Figure 8C**, **Table 3**). GO analysis also revealed five negatively correlated categories: Complement Activation, Antigen Receptor Mediated Signaling Pathway, B Cell Receptor Signaling Pathway, Antigen Binding and Immunoglobulin Complex (**Figure 8D**, **Table 3**).

**Table 2:** Signaling pathways most significantly correlated with VGF expression based on their NES and FDR by KEGG.

	KEGG ID	NES	pvalue	FDR
Positive	REACTOME_HDACS_DEACETYLATE_HISTONES	2.363	< 0.001	< 0.001
	REACTOME_SIRT1_NEGATIVELY_REGULATES_RRNA_EXPRESSION	2.286	< 0.001	< 0.001
	REACTOME_HCMV_LATE_EVENTS	2.268	< 0.001	< 0.001
	REACTOME_CONDENSATION_OF_PROPHASE_CHROMOSOMES	2.266	< 0.001	< 0.001
	REACTOME_PRC2_METHYLATES_HISTONES_AND_DNA	2.264	< 0.001	< 0.001
Negative	REACTOME_FCERI_MEDIATED_MAPK_ACTIVATION	-2.865	< 0.001	< 0.001
	REACTOME_ROLE_OF_PHOSPHOLIPIDS_IN_PHAGOCYTOSIS	-2.875	< 0.001	< 0.001
	REACTOME_ANTIGEN_ACTIVATES_B_CELL_RECEPTOR_BCR_LEADING_TO_GENERATION_OF_SECOND_MESSENGERS	-2.885	< 0.001	< 0.001
	REACTOME_PARASITE_INFECTION	-2.888	< 0.001	< 0.001
	REACTOME_FCERI_MEDIATED_CA_2_MOBILIZATION	-2.897	< 0.001	< 0.001

**Table 3:** Signaling pathways most significantly correlated with VGF expression based on their NES and FDR by GO.

	GO ID	NES	P value	FDR
Positive	GOBP_DIENCEPHALON_DEVELOPMENT	2.294	< 0.001	< 0.001
	GOBP_CELL_DIFFERENTIATION_IN_SPINAL_CORD	2.263	< 0.001	< 0.001
	GOBP_SPINAL_CORD_DEVELOPMENT	2.251	< 0.001	< 0.001
	GOBP_NEURON_FATE_COMMITMENT	2.202	< 0.001	< 0.001
	GOMF_STRUCTURAL_CONSTITUENT_OF_CHROMATIN	2.187	< 0.001	< 0.001
Negative	GOBP_COMPLEMENT_ACTIVATION	-2.878	< 0.001	< 0.001
	GOBP_ANTIGEN_RECEPTOR_MEDIATED_SIGNALING_PATHWAY	-2.889	< 0.001	< 0.001
	GOBP_B_CELL_RECEPTOR_SIGNALING_PATHWAY	-2.891	< 0.001	< 0.001
	GOMF_ANTIGEN_BINDING	-2.979	< 0.001	< 0.001
	GOCC_IMMUNOGLOBULIN_COMPLEX	-3.092	< 0.001	< 0.001

GO is primarily used to describe the biological roles of genes and proteins, while KEGG focuses on metabolic pathways, cellular signaling, and gene regulatory networks in organisms. These tools help researchers understand the specific biological functions and phenotypes associated with gene expression profiles, providing important insights for functional annotation and biological interpretation.

### The correlation between VGF expression and immune infiltration

**Figure 9:**
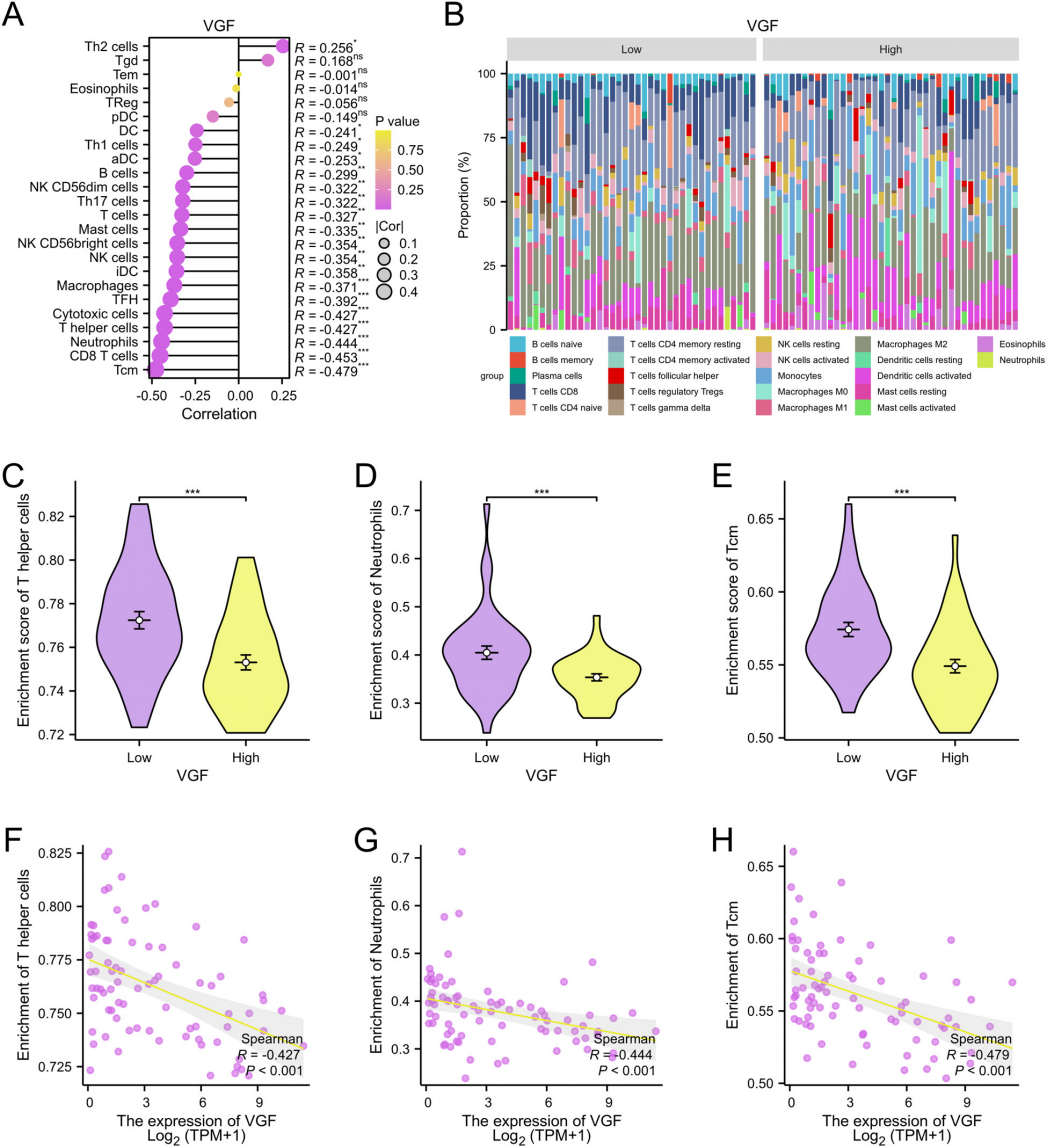
The expression level of VGF was associated with immune infiltration. **(A)** Correlations were observed between the relative abundances of 24 immune cells and VGF expression level. **(B)** Overlaying bar charts to demonstrate the differential data distribution of immune infiltration results between VGF high and low expression groups. **(C-H)** Violin Plots and correlation diagrams demonstrated the differences in T helper cells, Neutrophils cells and T central memory cells infiltration levels between the VGF-high and low groups. ***P<0.001.

The correlation between VGF expression level and immune cell infiltration level, as quantified by ssGSEA, was analyzed using Spearman correlation. VGF expression was found to have a positive correlation with the abundance of Th2 cells, and a negative correlation with the abundance of Neutrophils cells, CD8 T cells, and T central memory cells, among others (**Figure 9A**). Furthermore, we aimed to investigate potential variations in the tumor immune microenvironment between ACC patients with elevated levels of VGF and those with low levels. We have created Overlaying bar charts illustrating the disparity in immune infiltration findings between high and low expression groups (median) of the VGF molecule (**Figure 9B**).

Violin plots display the immune enrichment score between VGF high and low expression groups. Violin plots demonstrated the differences in T helper cells, Neutrophils cells and T central memory cells infiltration levels between the VGF-high and low groups (p < 0.001) (**Figures 9C-E**). Correlation diagrams indicate that the expression of VGF is negatively correlated with the levels of T helper cells (R = -0.427, p < 0.001 by Spearman), Neutrophils cells (R = -0.444, p < 0.001 by Spearman), and T central memory cells (R = -0.479, p < 0.001 by Spearman) (**Figures 9F-H**). These findings demonstrated that VGF may have a distinct function in immune infiltration in ACC.

## Discussion

ACC, a rare, aggressive cancer with poor prognosis, is treated with surgery, chemotherapy, and radiotherapy, each with limitations. Surgery is the only cure but poses high recurrence risk and low five-year survival rates. Incomplete resection, especially with positive margins or advanced disease, is a significant challenge. For advanced or metastatic ACC, chemotherapy is often used as a second-line treatment. The most commonly used chemotherapeutic agents include mitotane (an adrenal cytotoxic drug) combined with platinum-based chemotherapy (etoposide, doxorubicin, and cisplatin) (23, 24). However, the effectiveness of chemotherapy in ACC is limited, with poor response rates and frequent disease progression (25). Radiotherapy, an adjunctive treatment for unresectable or recurrent ACC, has uncertain efficacy and significant side effects (26). ACC is challenging to treat due to its aggression, high recurrence, and limited options. Better biomarkers are urgently needed for predicting response, guiding decisions, and identifying beneficial treatments.

The potential role of various biomarkers in the prognosis and treatment of ACC has been a subject of extensive research over the past decades (9, 27). VGF is predominantly known for its role in neuroendocrine regulation but has increasingly been implicated in cancer biology (28, 29). VGF was highly expressed in the nervous system, hypothalamus, and pituitary gland (30, 31). Primary carcinoma of the adrenal cortex is a rare and aggressive form of cancer. It accounts for approximately 0.02% of all cancers (32). The clinical presentation of this type of cancer can vary greatly. It may be non-functioning and asymptomatic or functioning, producing excess hormones like cortisol, androgens, or aldosterone, leading to overt clinical syndromes such as Cushing’s syndrome (32, 33). The estimated incidence rate is around 0.72 cases per million per year, with the disease contributing to 0.2% of all cancer-related deaths in the United States (34). The identification of reliable prognostic biomarkers and novel therapeutic targets is critical for improving the management and prognosis of ACC patients.

In the present study, we integrated VGF expression and prognostic values in ACC using TCGA, GTEx, GEPIA, MethSurv databases and R. VGF expression was Increased in tumor tissues compared with that in normal samples. Analysis of survival data from the TCGA database revealed that decreased VGF expression is correlated with poor prognosis and VGF expression is an independent factor for prognosis in ACC patients. ACC patients with high VGF expression are more likely to present a more advanced stage and lymphovascular invasion than those with high VGF expression. The GSEA was performed to uncover the potential biological function of VGF in ACC. GO term and KEGG pathway analysis revealed high levels of VGF correlated with the leukocyte mediated immunity, positive regulation of leukocyte activation, Cytokine-cytokine receptor interaction, and P13K-Akt signaling pathway.

Currently, a large number of scholars are focusing their research on the field of VGF in Alzheimer’s disease and neurocognition (35, 36). In recent years, VGF has been found to promote tumor metastasis and poor prognosis in breast and lung cancer (37, 38). VGF has been shown to regulate key signaling pathways involved in cell survival, proliferation, and migration, such as the PI3K/Akt pathway, which is frequently dysregulated in ACC and other malignancies (39, 40). The activation of the PI3K/Akt pathway has been implicated in promoting tumor cell proliferation and resistance to apoptosis, contributing to the aggressive nature of ACC. By regulating this pathway, VGF may directly influence tumor cell survival and resistance to treatment, further supporting its role in ACC progression.In addition, scholars have identified a CpG Island Methylator Phenotype in Adrenocortical Carcinoma, indicating the presence of hypermethylated adrenocortical carcinomas, which have a poorer prognosis (41). Researchers have discovered several gene mutations and alterations in DNA methylation in ACC samples with molecular subtypes linked to a worse prognosis. These genetic changes may impact the normal functioning and expression of genes, which in turn contributes to the development and advancement of tumors (42). The differential methylation patterns observed in this study provide insights into the epigenetic mechanisms that may contribute to ACC development and progression.

Immune infiltration in ACC is a hot topic at present. Understanding immune infiltrating cells is conducive to the development of immunotherapy for ACC. The correlation between VGF expression and diverse immune infiltration levels in ACC was analyzed in this study. Our data revealed that the expression of VGF was strongly correlated with infiltration levels of Th2 cells, T helper cells, Neutrophils, CD8 T cells. Adequate T cells infiltration is usually associated with a better prognosis (43). It is part of the immune system’s involvement in resisting pathogens, eliminating abnormal cells, and regulating tissue repair processes. Studies have shown that in the tumor microenvironment, patients with fewer infiltrating Th1 cells and more Th2 cells have a significantly worse prognosis (44). This is consistent with the immune infiltration of VGF in ACC as studied by us. The expression of VGF in the tumor immune microenvironment of ACC is negatively correlated with Th1 cells and T cells, providing evidence that high expression of VGF in ACC patients is associated with poor prognosis. The correlation between VGF expression and immune cell marker indicated a key role for VGF in regulating tumor immunology in ACC.

Admittedly, there were several limitations in this study. We only used public databases for analysis and the results need to be verified with more in experimental study. The findings only analyzed noticeable features without deeply exploring the mechanism. Although TCGA provides a comprehensive dataset, the relatively rare nature of ACC limits the sample size available for analysis. Therefore, more research is needed to confirm VGF efficacy and develop ACC immunotherapy in the future.

## Conclusion

VGF is the first neuropeptide precursor to be shown to correlate with immune infiltrates, survival prognosis, and tumor progression in ACC. VGF could be a potential biomarker for poor prognosis in ACC and may have a role in immune infiltration. VGF and related genes could potentially be targeted for immunotherapy in ACC. By addressing the limitations mentioned and exploring the future directions proposed, future research can utilize our findings to better comprehend the role of VGF in ACC. This has the potential to result in improved and personalized management strategies for patients dealing with this difficult cancer.

## Data Availability

The original contributions presented in the study are included in the article/**Supplementary Material**. Further inquiries can be directed to the corresponding author. https://zenodo.org/records/15081490.
