# Supplementary material for: A new predictive factor VGF based on IHC experiments, gene pathways and molecular functional groups for tumor immune microenvironment and prognosis of adrenocortical carcinoma
Source: Front Immunol. 2025 Apr 17;16:1542780. doi: 10.3389/fimmu.2025.1542780 (PMC12043488; doi:10.3389/fimmu.2025.1542780)
Supplement: Supplementary file 1 [file Table1.docx]

| Characteristics | Low expression of VGF | High expression of VGF | P value |
| --- | --- | --- | --- |
| n | 39 | 40 |  |
| Pathologic T stage, n (%) |  |  | 0.379 |
| T1 | 7 (9.1%) | 2 (2.6%) |  |
| T2 | 20 (26%) | 22 (28.6%) |  |
| T3 | 4 (5.2%) | 4 (5.2%) |  |
| T4 | 8 (10.4%) | 10 (13%) |  |
| Pathologic N stage, n (%) |  |  | 0.030 |
| N0 | 38 (49.4%) | 30 (39%) |  |
| N1 | 1 (1.3%) | 8 (10.4%) |  |
| Clinical M stage, n (%) |  |  | 0.135 |
| M0 | 34 (44.2%) | 28 (36.4%) |  |
| M1 | 5 (6.5%) | 10 (13%) |  |
| Pathologic stage, n (%) |  |  | 0.216 |
| Stage I | 7 (9.1%) | 2 (2.6%) |  |
| Stage II | 19 (24.7%) | 18 (23.4%) |  |
| Stage III | 8 (10.4%) | 8 (10.4%) |  |
| Stage IV | 5 (6.5%) | 10 (13%) |  |
| Gender, n (%) |  |  | 0.748 |
| Female | 23 (29.1%) | 25 (31.6%) |  |
| Male | 16 (20.3%) | 15 (19%) |  |
| Age, n (%) |  |  | 0.214 |
| <= 50 | 23 (29.1%) | 18 (22.8%) |  |
| > 50 | 16 (20.3%) | 22 (27.8%) |  |
| Tumor status, n (%) |  |  | < 0.001 |
| Tumor free | 28 (36.4%) | 11 (14.3%) |  |
| With tumor | 10 (13%) | 28 (36.4%) |  |
| Primary therapy outcome, n (%) |  |  | 0.085 |
| PD | 5 (7.5%) | 13 (19.4%) |  |
| SD | 1 (1.5%) | 1 (1.5%) |  |
| PR | 1 (1.5%) | 0 (0%) |  |
| CR | 28 (41.8%) | 18 (26.9%) |  |
| Race, n (%) |  |  | 0.232 |
| Asian | 1 (1.5%) | 0 (0%) |  |
| Black or African American | 1 (1.5%) | 0 (0%) |  |
| White | 31 (45.6%) | 35 (51.5%) |  |
| Residual tumor, n (%) |  |  | 0.146 |
| R0 | 30 (42.9%) | 25 (35.7%) |  |
| R1 | 4 (5.7%) | 2 (2.9%) |  |
| R2 | 2 (2.9%) | 7 (10%) |  |
| Laterality, n (%) |  |  | 0.314 |
| Left | 20 (25.3%) | 25 (31.6%) |  |
| Right | 19 (24.1%) | 15 (19%) |  |
| Mitotane therapy, n (%) |  |  | 0.028 |
| No | 17 (22.7%) | 9 (12%) |  |
| Yes | 19 (25.3%) | 30 (40%) |  |
| Radiation therapy, n (%) |  |  | 0.783 |
| No | 29 (38.2%) | 30 (39.5%) |  |
| Yes | 9 (11.8%) | 8 (10.5%) |  |
| OS event, n (%) |  |  | < 0.001 |
| Alive | 33 (41.8%) | 18 (22.8%) |  |
| Dead | 6 (7.6%) | 22 (27.8%) |  |
| DSS event, n (%) |  |  | < 0.001 |
| No | 33 (42.9%) | 18 (23.4%) |  |
| Yes | 5 (6.5%) | 21 (27.3%) |  |
| PFI event, n (%) |  |  | < 0.001 |
| No | 28 (35.4%) | 10 (12.7%) |  |
| Yes | 11 (13.9%) | 30 (38%) |  |
