# Supplementary material for: A new predictive factor VGF based on IHC experiments, gene pathways and molecular functional groups for tumor immune microenvironment and prognosis of adrenocortical carcinoma
Source: Front Immunol. 2025 Apr 17;16:1542780. doi: 10.3389/fimmu.2025.1542780 (PMC12043488; doi:10.3389/fimmu.2025.1542780)
Supplement: Supplementary file 2 [file Table2.docx]

| Ontology | ID | Description | GeneRatio | pvalue | p.adjust | qvalue |
| --- | --- | --- | --- | --- | --- | --- |
| BP | GO:0002443 | leukocyte mediated immunity | 166/2187 | 3.52E-44 | 2.01E-40 | 1.5E-40 |
| BP | GO:0050867 | positive regulation of cell activation | 159/2187 | 1.39E-42 | 2.65E-39 | 1.97E-39 |
| BP | GO:0002696 | positive regulation of leukocyte activation | 156/2187 | 8.61E-43 | 2.46E-39 | 1.83E-39 |
| BP | GO:0002764 | immune response-regulating signaling pathway | 145/2187 | 1.91E-28 | 7.25E-26 | 5.4E-26 |
| BP | GO:0051251 | positive regulation of lymphocyte activation | 138/2187 | 3.92E-38 | 5.59E-35 | 4.17E-35 |
| CC | GO:0009897 | external side of plasma membrane | 182/2310 | 9.71E-55 | 5.54E-52 | 4.28E-52 |
| CC | GO:0062023 | collagen-containing extracellular matrix | 113/2310 | 5.04E-17 | 5.74E-15 | 4.44E-15 |
| CC | GO:0098802 | plasma membrane signaling receptor complex | 100/2310 | 1.26E-21 | 1.8E-19 | 1.39E-19 |
| CC | GO:0043025 | neuronal cell body | 97/2310 | 7.32E-08 | 2.78E-06 | 2.15E-06 |
| CC | GO:0098793 | presynapse | 95/2310 | 7.17E-07 | 1.7E-05 | 1.32E-05 |
| MF | GO:0048018 | receptor ligand activity | 122/2168 | 2.23E-16 | 4.59E-14 | 3.72E-14 |
| MF | GO:0030546 | signaling receptor activator activity | 122/2168 | 7.07E-16 | 1.21E-13 | 9.83E-14 |
| MF | GO:0015267 | channel activity | 109/2168 | 2.13E-11 | 1.88E-09 | 1.53E-09 |
| MF | GO:0022803 | passive transmembrane transporter activity | 109/2168 | 2.43E-11 | 1.92E-09 | 1.56E-09 |
| MF | GO:0005216 | ion channel activity | 98/2168 | 3.02E-10 | 1.63E-08 | 1.33E-08 |
| KEGG | hsa04060 | Cytokine-cytokine receptor interaction | 100/1002 | 4.17E-23 | 1.32E-20 | 9.6E-21 |
| KEGG | hsa04080 | Neuroactive ligand-receptor interaction | 97/1002 | 1.24E-14 | 9.81E-13 | 7.13E-13 |
| KEGG | hsa04151 | PI3K-Akt signaling pathway | 72/1002 | 7.14E-06 | 0.000113 | 8.23E-05 |
| KEGG | hsa04062 | Chemokine signaling pathway | 60/1002 | 1.71E-12 | 1.08E-10 | 7.87E-11 |
| KEGG | hsa04020 | Calcium signaling pathway | 57/1002 | 4.33E-07 | 8.58E-06 | 6.24E-06 |
