# Supplementary figures and images for: A new predictive factor VGF based on IHC experiments, gene pathways and molecular functional groups for tumor immune microenvironment and prognosis of adrenocortical carcinoma
Source: Front Immunol. 2025 Apr 17;16:1542780. doi: 10.3389/fimmu.2025.1542780 (PMC12043488; doi:10.3389/fimmu.2025.1542780)

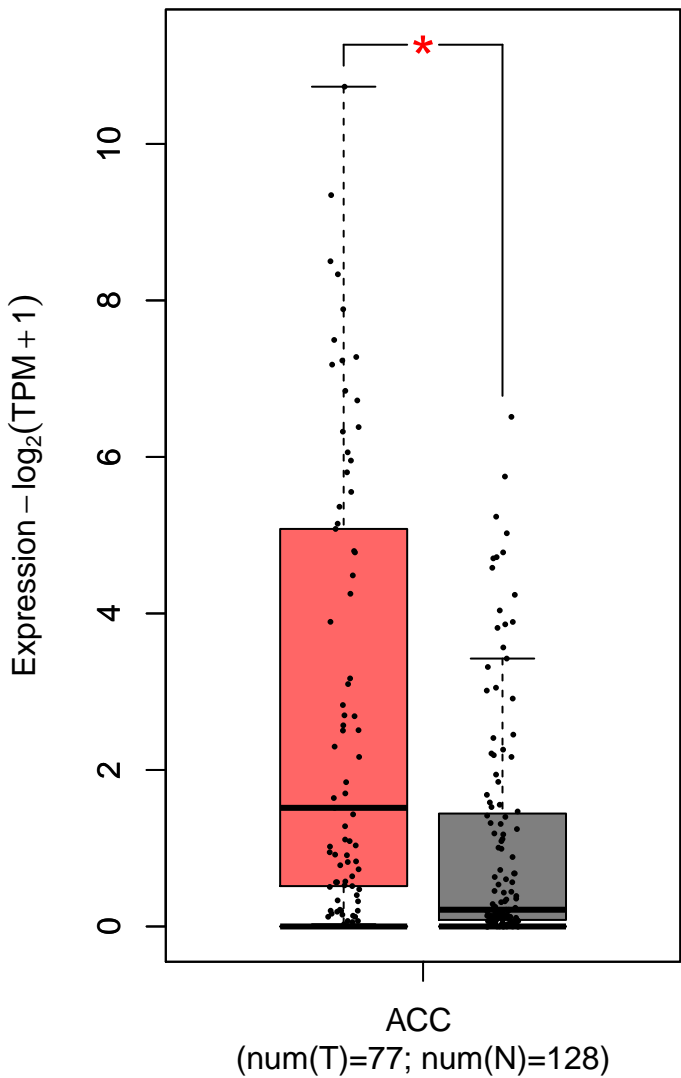

Supplement: Supplementary file 3 [file Image1.pdf]
